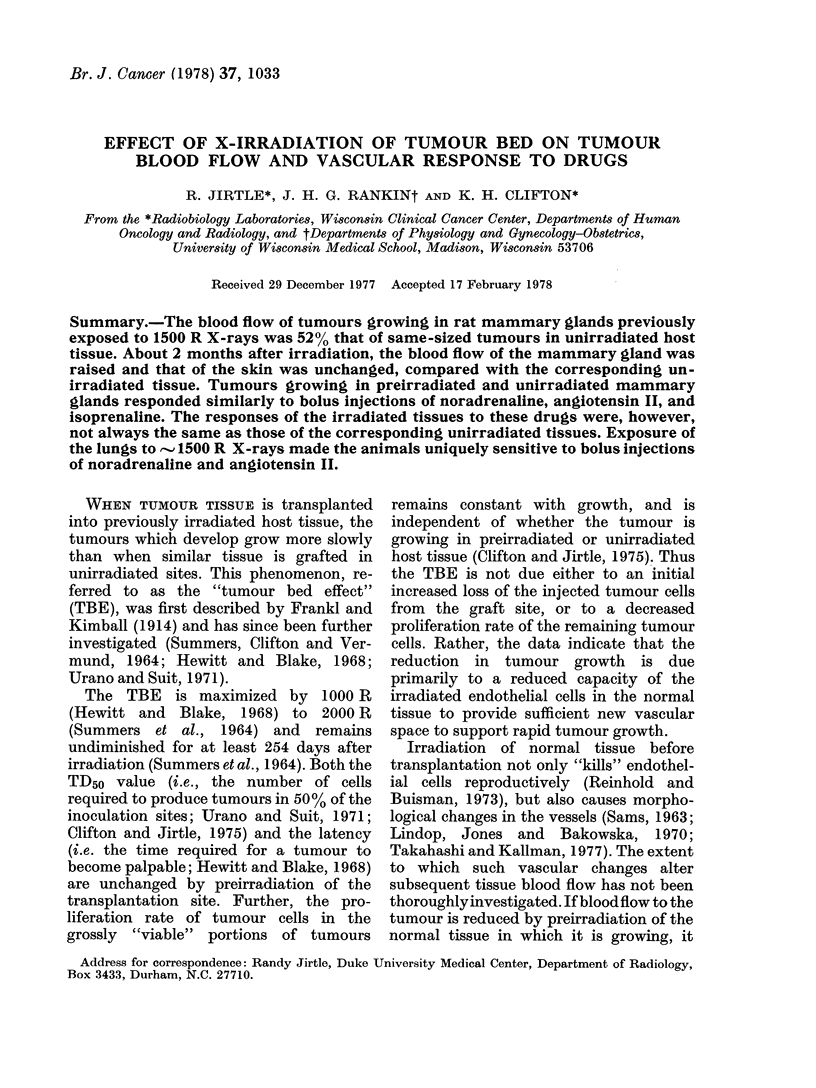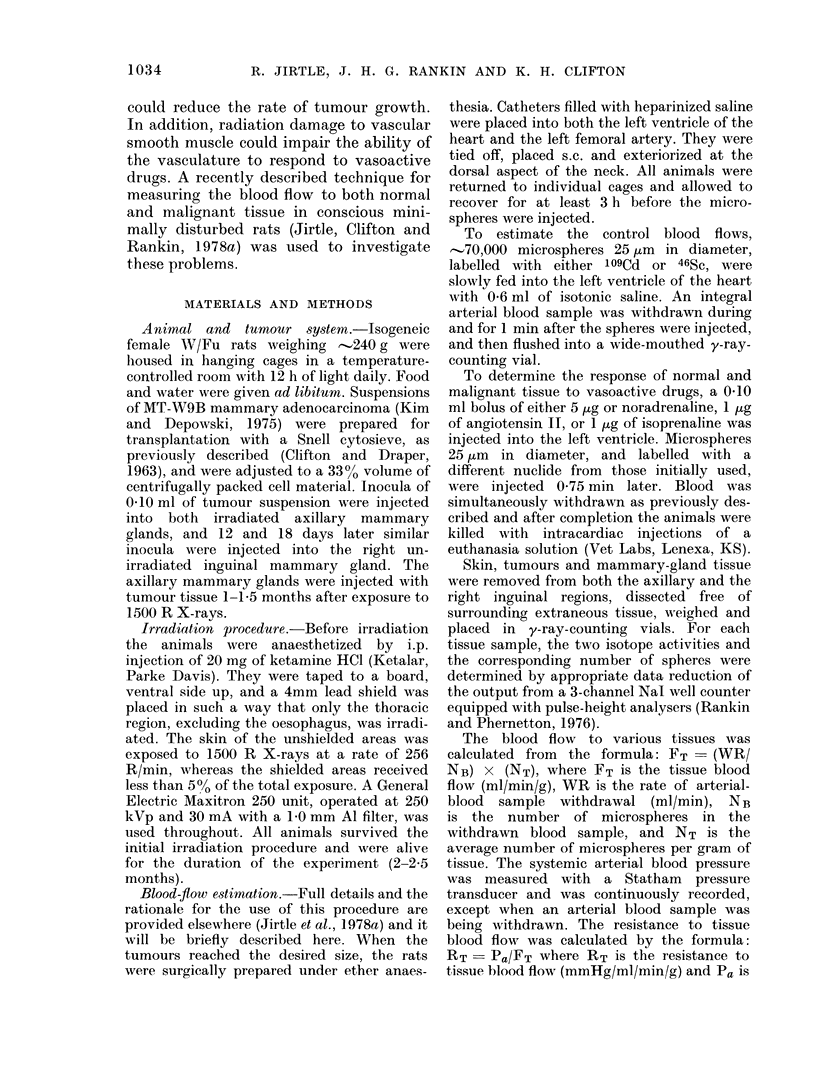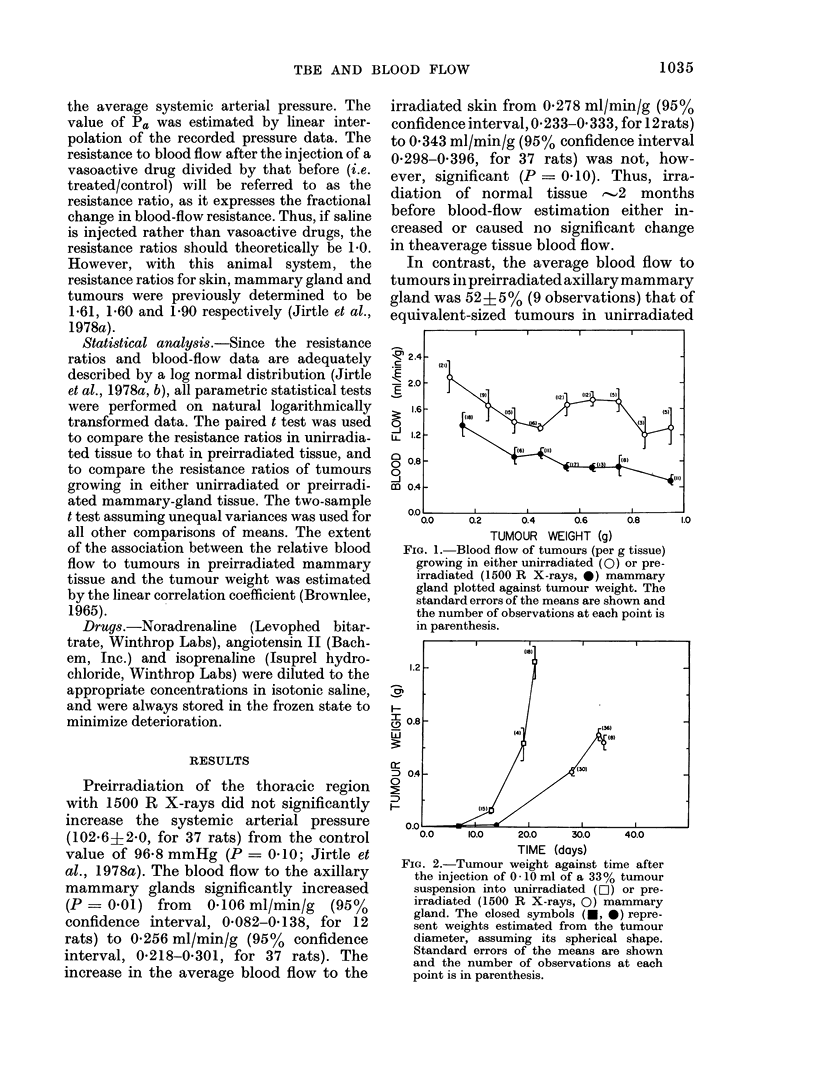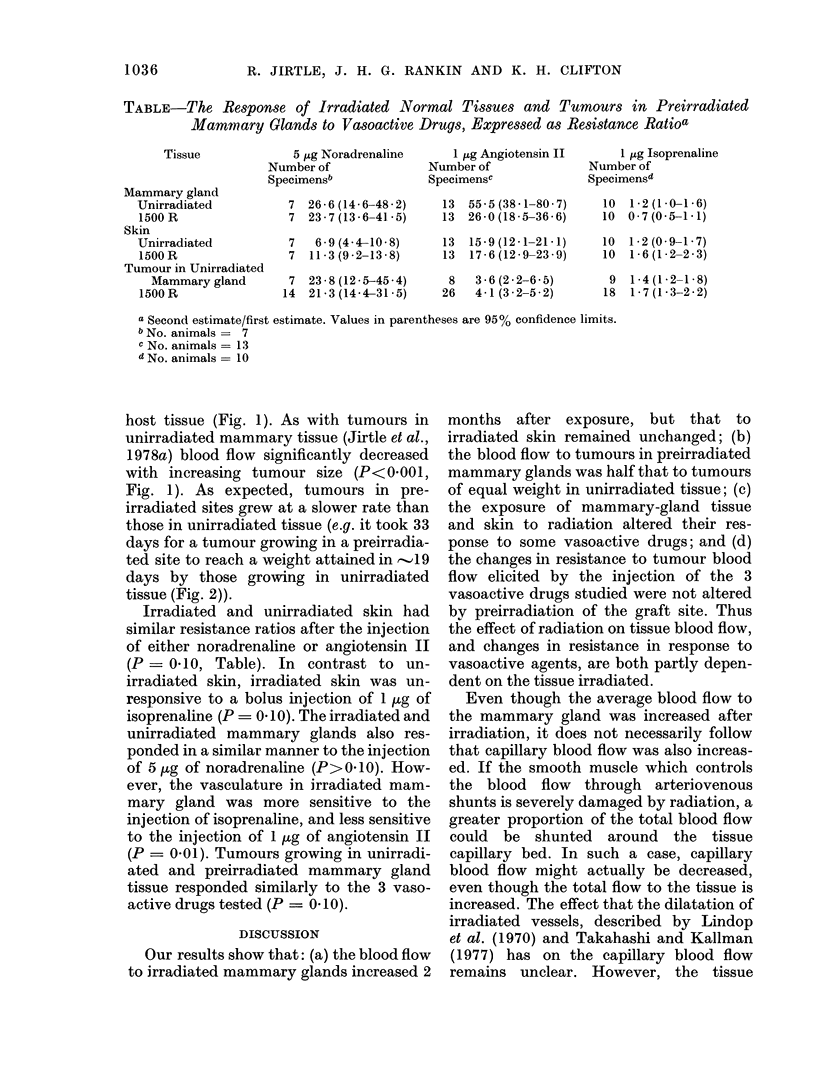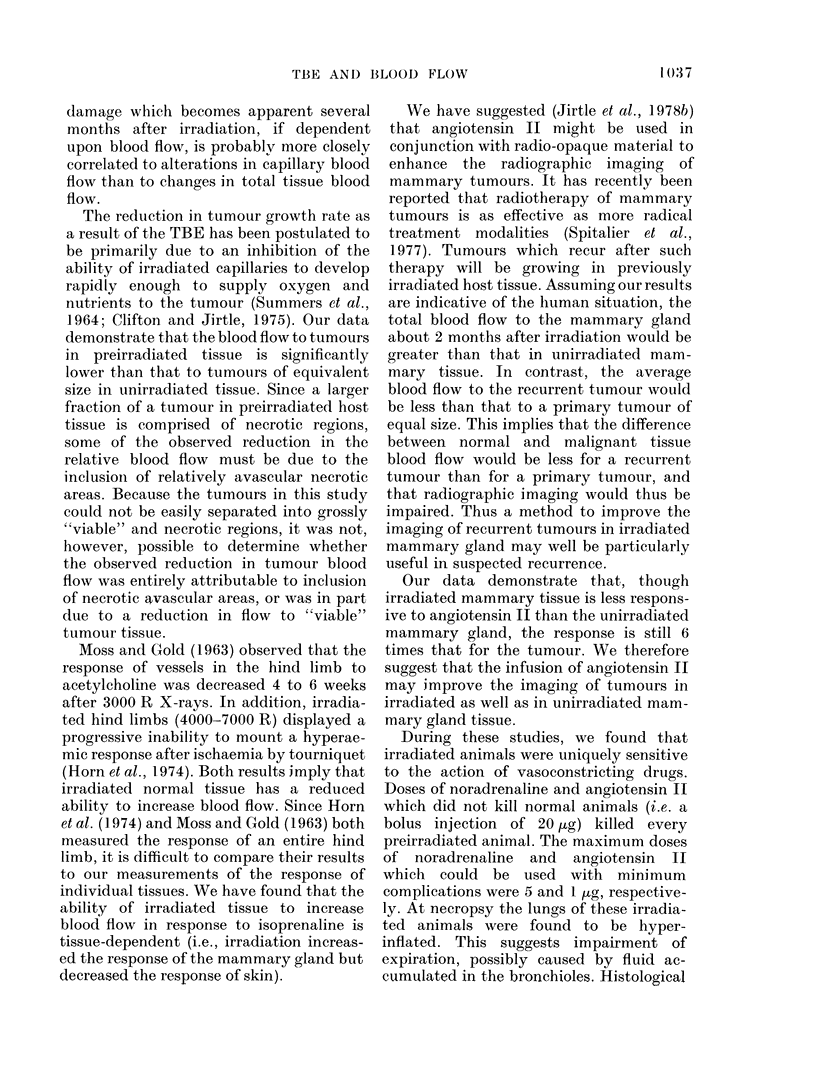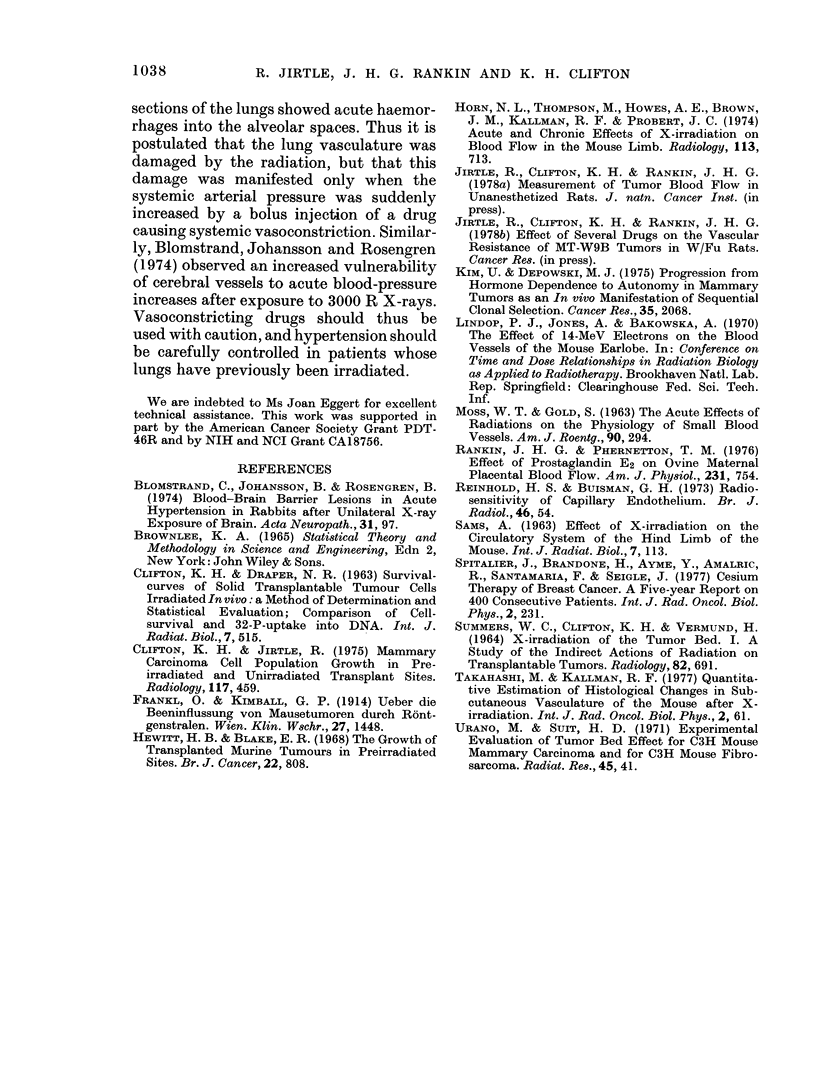# Effect of x-irradiation of tumour bed on tumour blood flow and vascular response to drugs.

**DOI:** 10.1038/bjc.1978.150

**Published:** 1978-06

**Authors:** R. Jirtle, J. H. Rankin, K. H. Clifton

## Abstract

The blood flow of tumours growing in rat mammary glands previously exposed to 1500 R X-rays was 52% that of same-sized tumours in unirradiated host tissue. About 2 months after irradiation, the blood flow of the mammary gland was raised and that of the skin was unchanged, compared with the corresponding unirradiated tissue. Tumours growing in preirradiated and unirradiated mammary glands responded similarly to bolus injections of noradrenaline, angiotensin II, and isoprenaline. The responses of the irradiated tissues to these drugs were, however, not always the same as those of the corresponding unirradiated tissues. Exposure of the lungs to approximately 1500 R X-rays made the animals uniquely sensitive to bolus injections of noradrenaline and angiotensin II.


*Br. J. Cancer* (1978) **37**, 1033

### EFFECT OF X-IRRADIATION OF TUMOUR BED ON TUMOUR BLOOD FLOW AND VASCULAR RESPONSE TO DRUGS

R. JIRTLE*, J. H. G. RANKIN† AND K. H. CLIFTON*

*From the *Radiobiology Laboratories, Wisconsin Clinical Cancer Center, Departments of Human Oncology and Radiology, and †Departments of Physiology and Gynecology–Obstetrics, University of Wisconsin Medical School, Madison, Wisconsin* 53706

Received 29 December 1977 Accepted 17 February 1978

**Summary.—The blood flow of tumours growing in rat mammary glands previously exposed to 1500 R X-rays was 52% that of same-sized tumours in unirradiated host tissue. About 2 months after irradiation, the blood flow of the mammary gland was raised and that of the skin was unchanged, compared with the corresponding unirradiated tissue. Tumours growing in preirradiated and unirradiated mammary glands responded similarly to bolus injections of noradrenaline, angiotensin II, and isoprenaline. The responses of the irradiated tissues to these drugs were, however, not always the same as those of the corresponding unirradiated tissues. Exposure of the lungs to ~1500 R X-rays made the animals uniquely sensitive to bolus injections of noradrenaline and angiotensin II.**

WHEN TUMOUR TISSUE is transplanted into previously irradiated host tissue, the tumours which develop grow more slowly than when similar tissue is grafted in unirradiated sites. This phenomenon, referred to as the "tumour bed effect" (TBE), was first described by Frankl and Kimball (1914) and has since been further investigated (Summers, Clifton and Vermund, 1964; Hewitt and Blake, 1968; Urano and Suit, 1971).

The TBE is maximized by 1000 R (Hewitt and Blake, 1968) to 2000 R (Summers *et al.*, 1964) and remains undiminished for at least 254 days after irradiation (Summers *et al.*, 1964). Both the $TD_{50}$ value (*i.e.*, the number of cells required to produce tumours in 50% of the inoculation sites; Urano and Suit, 1971; Clifton and Jirtle, 1975) and the latency (*i.e.* the time required for a tumour to become palpable; Hewitt and Blake, 1968) are unchanged by preirradiation of the transplantation site. Further, the proliferation rate of tumour cells in the grossly "viable" portions of tumours remains constant with growth, and is independent of whether the tumour is growing in preirradiated or unirradiated host tissue (Clifton and Jirtle, 1975). Thus the TBE is not due either to an initial increased loss of the injected tumour cells from the graft site, or to a decreased proliferation rate of the remaining tumour cells. Rather, the data indicate that the reduction in tumour growth is due primarily to a reduced capacity of the irradiated endothelial cells in the normal tissue to provide sufficient new vascular space to support rapid tumour growth.

Irradiation of normal tissue before transplantation not only "kills" endothelial cells reproductively (Reinhold and Buisman, 1973), but also causes morphological changes in the vessels (Sams, 1963; Lindop, Jones and Bakowska, 1970; Takahashi and Kallman, 1977). The extent to which such vascular changes alter subsequent tissue blood flow has not been thoroughly investigated. If blood flow to the tumour is reduced by preirradiation of the normal tissue in which it is growing, it

Address for correspondence: Randy Jirtle, Duke University Medical Center, Department of Radiology, Box 3433, Durham, N.C. 27710.

could reduce the rate of tumour growth. In addition, radiation damage to vascular smooth muscle could impair the ability of the vasculature to respond to vasoactive drugs. A recently described technique for measuring the blood flow to both normal and malignant tissue in conscious minimally disturbed rats (Jirtle, Clifton and Rankin, 1978*a*) was used to investigate these problems.

### MATERIALS AND METHODS

*Animal and tumour system.*—Isogeneic female W/Fu rats weighing ~240 g were housed in hanging cages in a temperature-controlled room with 12 h of light daily. Food and water were given *ad libitum*. Suspensions of MT-W9B mammary adenocarcinoma (Kim and Depowski, 1975) were prepared for transplantation with a Snell cytosieve, as previously described (Clifton and Draper, 1963), and were adjusted to a 33% volume of centrifugally packed cell material. Inocula of 0·10 ml of tumour suspension were injected into both irradiated axillary mammary glands, and 12 and 18 days later similar inocula were injected into the right unirradiated inguinal mammary gland. The axillary mammary glands were injected with tumour tissue 1–1·5 months after exposure to 1500 R X-rays.

*Irradiation procedure.*—Before irradiation the animals were anaesthetized by i.p. injection of 20 mg of ketamine HCl (Ketalar, Parke Davis). They were taped to a board, ventral side up, and a 4mm lead shield was placed in such a way that only the thoracic region, excluding the oesophagus, was irradiated. The skin of the unshielded areas was exposed to 1500 R X-rays at a rate of 256 R/min, whereas the shielded areas received less than 5% of the total exposure. A General Electric Maxitron 250 unit, operated at 250 kVp and 30 mA with a 1·0 mm Al filter, was used throughout. All animals survived the initial irradiation procedure and were alive for the duration of the experiment (2–2·5 months).

*Blood-flow estimation.*—Full details and the rationale for the use of this procedure are provided elsewhere (Jirtle *et al.*, 1978*a*) and it will be briefly described here. When the tumours reached the desired size, the rats were surgically prepared under ether anaesthesia. Catheters filled with heparinized saline were placed into both the left ventricle of the heart and the left femoral artery. They were tied off, placed s.c. and exteriorized at the dorsal aspect of the neck. All animals were returned to individual cages and allowed to recover for at least 3 h before the microspheres were injected.

To estimate the control blood flows, ~70,000 microspheres 25 μm in diameter, labelled with either $^{109}Cd$ or $^{46}Sc$, were slowly fed into the left ventricle of the heart with 0·6 ml of isotonic saline. An integral arterial blood sample was withdrawn during and for 1 min after the spheres were injected, and then flushed into a wide-mouthed γ-ray-counting vial.

To determine the response of normal and malignant tissue to vasoactive drugs, a 0·10 ml bolus of either 5 μg or noradrenaline, 1 μg of angiotensin II, or 1 μg of isoprenaline was injected into the left ventricle. Microspheres 25 μm in diameter, and labelled with a different nuclide from those initially used, were injected 0·75 min later. Blood was simultaneously withdrawn as previously described and after completion the animals were killed with intracardiac injections of a euthanasia solution (Vet Labs, Lenexa, KS).

Skin, tumours and mammary-gland tissue were removed from both the axillary and the right inguinal regions, dissected free of surrounding extraneous tissue, weighed and placed in γ-ray-counting vials. For each tissue sample, the two isotope activities and the corresponding number of spheres were determined by appropriate data reduction of the output from a 3-channel NaI well counter equipped with pulse-height analysers (Rankin and Phernetton, 1976).

The blood flow to various tissues was calculated from the formula: $F_T = (WR/N_B) \times (N_T)$, where $F_T$ is the tissue blood flow (ml/min/g), WR is the rate of arterial-blood sample withdrawal (ml/min), $N_B$ is the number of microspheres in the withdrawn blood sample, and $N_T$ is the average number of microspheres per gram of tissue. The systemic arterial blood pressure was measured with a Statham pressure transducer and was continuously recorded, except when an arterial blood sample was being withdrawn. The resistance to tissue blood flow was calculated by the formula: $R_T = P_a/F_T$ where $R_T$ is the resistance to tissue blood flow (mmHg/ml/min/g) and $P_a$ is the average systemic arterial pressure. The value of $P_a$ was estimated by linear interpolation of the recorded pressure data. The resistance to blood flow after the injection of a vasoactive drug divided by that before (*i.e.* treated/control) will be referred to as the resistance ratio, as it expresses the fractional change in blood-flow resistance. Thus, if saline is injected rather than vasoactive drugs, the resistance ratios should theoretically be 1·0. However, with this animal system, the resistance ratios for skin, mammary gland and tumours were previously determined to be 1·61, 1·60 and 1·90 respectively (Jirtle *et al.*, 1978*a*).

*Statistical analysis.*—Since the resistance ratios and blood-flow data are adequately described by a log normal distribution (Jirtle *et al.*, 1978*a*, *b*), all parametric statistical tests were performed on natural logarithmically transformed data. The paired *t* test was used to compare the resistance ratios in unirradiated tissue to that in preirradiated tissue, and to compare the resistance ratios of tumours growing in either unirradiated or preirradiated mammary-gland tissue. The two-sample *t* test assuming unequal variances was used for all other comparisons of means. The extent of the association between the relative blood flow to tumours in preirradiated mammary tissue and the tumour weight was estimated by the linear correlation coefficient (Brownlee, 1965).

*Drugs.*—Noradrenaline (Levophed bitartrate, Winthrop Labs), angiotensin II (Bachem, Inc.) and isoprenaline (Isuprel hydrochloride, Winthrop Labs) were diluted to the appropriate concentrations in isotonic saline, and were always stored in the frozen state to minimize deterioration.

### RESULTS

Preirradiation of the thoracic region with 1500 R X-rays did not significantly increase the systemic arterial pressure (102·6±2·0, for 37 rats) from the control value of 96·8 mmHg ($P = 0{\cdot}10$; Jirtle *et al.*, 1978*a*). The blood flow to the axillary mammary glands significantly increased ($P = 0{\cdot}01$) from 0·106 ml/min/g (95% confidence interval, 0·082–0·138, for 12 rats) to 0·256 ml/min/g (95% confidence interval, 0·218–0·301, for 37 rats). The increase in the average blood flow to the irradiated skin from 0·278 ml/min/g (95% confidence interval, 0·233–0·333, for 12 rats) to 0·343 ml/min/g (95% confidence interval 0·298–0·396, for 37 rats) was not, however, significant ($P = 0{\cdot}10$). Thus, irradiation of normal tissue ~2 months before blood-flow estimation either increased or caused no significant change in theaverage tissue blood flow.

In contrast, the average blood flow to tumours in preirradiated axillary mammary gland was 52±5% (9 observations) that of equivalent-sized tumours in unirradiated

FIG. 1.—Blood flow of tumours (per g tissue) growing in either unirradiated (○) or preirradiated (1500 R X-rays, ●) mammary gland plotted against tumour weight. The standard errors of the means are shown and the number of observations at each point is in parenthesis.

FIG. 2.—Tumour weight against time after the injection of 0·10 ml of a 33% tumour suspension into unirradiated (□) or preirradiated (1500 R X-rays, ○) mammary gland. The closed symbols (■, ●) represent weights estimated from the tumour diameter, assuming its spherical shape. Standard errors of the means are shown and the number of observations at each point is in parenthesis.

TABLE—*The Response of Irradiated Normal Tissues and Tumours in Preirradiated Mammary Glands to Vasoactive Drugs, Expressed as Resistance Ratio*[a]

| Tissue | 5 μg Noradrenaline Number of Specimens[b] | | 1 μg Angiotensin II Number of Specimens[c] | | 1 μg Isoprenaline Number of Specimens[d] | |
|---|---|---|---|---|---|---|
| Mammary gland | | | | | | |
| Unirradiated | 7 | 26·6 (14·6–48·2) | 13 | 55·5 (38·1–80·7) | 10 | 1·2 (1·0–1·6) |
| 1500 R | 7 | 23·7 (13·6–41·5) | 13 | 26·0 (18·5–36·6) | 10 | 0·7 (0·5–1·1) |
| Skin | | | | | | |
| Unirradiated | 7 | 6·9 (4·4–10·8) | 13 | 15·9 (12·1–21·1) | 10 | 1·2 (0·9–1·7) |
| 1500 R | 7 | 11·3 (9·2–13·8) | 13 | 17·6 (12·9–23·9) | 10 | 1·6 (1·2–2·3) |
| Tumour in Unirradiated Mammary gland | 7 | 23·8 (12·5–45·4) | 8 | 3·6 (2·2–6·5) | 9 | 1·4 (1·2–1·8) |
| 1500 R | 14 | 21·3 (14·4–31·5) | 26 | 4·1 (3·2–5·2) | 18 | 1·7 (1·3–2·2) |

[a] Second estimate/first estimate. Values in parentheses are 95% confidence limits.
[b] No. animals = 7
[c] No. animals = 13
[d] No. animals = 10

host tissue (Fig. 1). As with tumours in unirradiated mammary tissue (Jirtle *et al.*, 1978*a*) blood flow significantly decreased with increasing tumour size ($P<0·001$, Fig. 1). As expected, tumours in preirradiated sites grew at a slower rate than those in unirradiated tissue (*e.g.* it took 33 days for a tumour growing in a preirradiated site to reach a weight attained in ~19 days by those growing in unirradiated tissue (Fig. 2)).

Irradiated and unirradiated skin had similar resistance ratios after the injection of either noradrenaline or angiotensin II ($P = 0·10$, Table). In contrast to unirradiated skin, irradiated skin was unresponsive to a bolus injection of 1 μg of isoprenaline ($P = 0·10$). The irradiated and unirradiated mammary glands also responded in a similar manner to the injection of 5 μg of noradrenaline ($P>0·10$). However, the vasculature in irradiated mammary gland was more sensitive to the injection of isoprenaline, and less sensitive to the injection of 1 μg of angiotensin II ($P = 0·01$). Tumours growing in unirradiated and preirradiated mammary gland tissue responded similarly to the 3 vasoactive drugs tested ($P = 0·10$).

### DISCUSSION

Our results show that: (a) the blood flow to irradiated mammary glands increased 2 months after exposure, but that to irradiated skin remained unchanged; (b) the blood flow to tumours in preirradiated mammary glands was half that to tumours of equal weight in unirradiated tissue; (c) the exposure of mammary-gland tissue and skin to radiation altered their response to some vasoactive drugs; and (d) the changes in resistance to tumour blood flow elicited by the injection of the 3 vasoactive drugs studied were not altered by preirradiation of the graft site. Thus the effect of radiation on tissue blood flow, and changes in resistance in response to vasoactive agents, are both partly dependent on the tissue irradiated.

Even though the average blood flow to the mammary gland was increased after irradiation, it does not necessarily follow that capillary blood flow was also increased. If the smooth muscle which controls the blood flow through arteriovenous shunts is severely damaged by radiation, a greater proportion of the total blood flow could be shunted around the tissue capillary bed. In such a case, capillary blood flow might actually be decreased, even though the total flow to the tissue is increased. The effect that the dilatation of irradiated vessels, described by Lindop *et al.* (1970) and Takahashi and Kallman (1977) has on the capillary blood flow remains unclear. However, the tissue damage which becomes apparent several months after irradiation, if dependent upon blood flow, is probably more closely correlated to alterations in capillary blood flow than to changes in total tissue blood flow.

The reduction in tumour growth rate as a result of the TBE has been postulated to be primarily due to an inhibition of the ability of irradiated capillaries to develop rapidly enough to supply oxygen and nutrients to the tumour (Summers *et al.*, 1964; Clifton and Jirtle, 1975). Our data demonstrate that the blood flow to tumours in preirradiated tissue is significantly lower than that to tumours of equivalent size in unirradiated tissue. Since a larger fraction of a tumour in preirradiated host tissue is comprised of necrotic regions, some of the observed reduction in the relative blood flow must be due to the inclusion of relatively avascular necrotic areas. Because the tumours in this study could not be easily separated into grossly "viable" and necrotic regions, it was not, however, possible to determine whether the observed reduction in tumour blood flow was entirely attributable to inclusion of necrotic avascular areas, or was in part due to a reduction in flow to "viable" tumour tissue.

Moss and Gold (1963) observed that the response of vessels in the hind limb to acetylcholine was decreased 4 to 6 weeks after 3000 R X-rays. In addition, irradiated hind limbs (4000–7000 R) displayed a progressive inability to mount a hyperaemic response after ischaemia by tourniquet (Horn *et al.*, 1974). Both results imply that irradiated normal tissue has a reduced ability to increase blood flow. Since Horn *et al.* (1974) and Moss and Gold (1963) both measured the response of an entire hind limb, it is difficult to compare their results to our measurements of the response of individual tissues. We have found that the ability of irradiated tissue to increase blood flow in response to isoprenaline is tissue-dependent (i.e., irradiation increased the response of the mammary gland but decreased the response of skin).

We have suggested (Jirtle *et al.*, 1978*b*) that angiotensin II might be used in conjunction with radio-opaque material to enhance the radiographic imaging of mammary tumours. It has recently been reported that radiotherapy of mammary tumours is as effective as more radical treatment modalities (Spitalier *et al.*, 1977). Tumours which recur after such therapy will be growing in previously irradiated host tissue. Assuming our results are indicative of the human situation, the total blood flow to the mammary gland about 2 months after irradiation would be greater than that in unirradiated mammary tissue. In contrast, the average blood flow to the recurrent tumour would be less than that to a primary tumour of equal size. This implies that the difference between normal and malignant tissue blood flow would be less for a recurrent tumour than for a primary tumour, and that radiographic imaging would thus be impaired. Thus a method to improve the imaging of recurrent tumours in irradiated mammary gland may well be particularly useful in suspected recurrence.

Our data demonstrate that, though irradiated mammary tissue is less responsive to angiotensin II than the unirradiated mammary gland, the response is still 6 times that for the tumour. We therefore suggest that the infusion of angiotensin II may improve the imaging of tumours in irradiated as well as in unirradiated mammary gland tissue.

During these studies, we found that irradiated animals were uniquely sensitive to the action of vasoconstricting drugs. Doses of noradrenaline and angiotensin II which did not kill normal animals (*i.e.* a bolus injection of 20 $\mu$g) killed every preirradiated animal. The maximum doses of noradrenaline and angiotensin II which could be used with minimum complications were 5 and 1 $\mu$g, respectively. At necropsy the lungs of these irradiated animals were found to be hyperinflated. This suggests impairment of expiration, possibly caused by fluid accumulated in the bronchioles. Histological sections of the lungs showed acute haemorrhages into the alveolar spaces. Thus it is postulated that the lung vasculature was damaged by the radiation, but that this damage was manifested only when the systemic arterial pressure was suddenly increased by a bolus injection of a drug causing systemic vasoconstriction. Similarly, Blomstrand, Johansson and Rosengren (1974) observed an increased vulnerability of cerebral vessels to acute blood-pressure increases after exposure to 3000 R X-rays. Vasoconstricting drugs should thus be used with caution, and hypertension should be carefully controlled in patients whose lungs have previously been irradiated.

We are indebted to Ms Joan Eggert for excellent technical assistance. This work was supported in part by the American Cancer Society Grant PDT-46R and by NIH and NCI Grant CA18756.

### REFERENCES

BLOMSTRAND, C., JOHANSSON, B. & ROSENGREN, B. (1974) Blood–Brain Barrier Lesions in Acute Hypertension in Rabbits after Unilateral X-ray Exposure of Brain. *Acta Neuropath.*, **31**, 97.

BROWNLEE, K. A. (1965) *Statistical Theory and Methodology in Science and Engineering*, Edn 2, New York: John Wiley & Sons.

CLIFTON, K. H. & DRAPER, N. R. (1963) Survival-curves of Solid Transplantable Tumour Cells Irradiated *In vivo*: a Method of Determination and Statistical Evaluation; Comparison of Cell-survival and 32-P-uptake into DNA. *Int. J. Radiat. Biol.*, **7**, 515.

CLIFTON, K. H. & JIRTLE, R. (1975) Mammary Carcinoma Cell Population Growth in Pre-irradiated and Unirradiated Transplant Sites. *Radiology*, **117**, 459.

FRANKL, O. & KIMBALL, G. P. (1914) Ueber die Beeninflussung von Mausetumoren durch Röntgenstralen. *Wien. Klin. Wschr.*, **27**, 1448.

HEWITT, H. B. & BLAKE, E. R. (1968) The Growth of Transplanted Murine Tumours in Preirradiated Sites. *Br. J. Cancer*, **22**, 808.

HORN, N. L., THOMPSON, M., HOWES, A. E., BROWN, J. M., KALLMAN, R. F. & PROBERT, J. C. (1974) Acute and Chronic Effects of X-irradiation on Blood Flow in the Mouse Limb. *Radiology*, **113**, 713.

JIRTLE, R., CLIFTON, K. H. & RANKIN, J. H. G. (1978*a*) Measurement of Tumor Blood Flow in Unanesthetized Rats. *J. natn. Cancer Inst.* (in press).

JIRTLE, R., CLIFTON, K. H. & RANKIN, J. H. G. (1978*b*) Effect of Several Drugs on the Vascular Resistance of MT-W9B Tumors in W/Fu Rats. *Cancer Res.* (in press).

KIM, U. & DEPOWSKI, M. J. (1975) Progression from Hormone Dependence to Autonomy in Mammary Tumors as an *In vivo* Manifestation of Sequential Clonal Selection. *Cancer Res.*, **35**, 2068.

LINDOP, P. J., JONES, A. & BAKOWSKA, A. (1970) The Effect of 14-MeV Electrons on the Blood Vessels of the Mouse Earlobe. In: *Conference on Time and Dose Relationships in Radiation Biology as Applied to Radiotherapy*. Brookhaven Natl. Lab. Rep. Springfield: Clearinghouse Fed. Sci. Tech. Inf.

MOSS, W. T. & GOLD, S. (1963) The Acute Effects of Radiations on the Physiology of Small Blood Vessels. *Am. J. Roentg.*, **90**, 294.

RANKIN, J. H. G. & PHERNETTON, T. M. (1976) Effect of Prostaglandin $E_2$ on Ovine Maternal Placental Blood Flow. *Am. J. Physiol.*, **231**, 754.

REINHOLD, H. S. & BUISMAN, G. H. (1973) Radiosensitivity of Capillary Endothelium. *Br. J. Radiol.*, **46**, 54.

SAMS, A. (1963) Effect of X-irradiation on the Circulatory System of the Hind Limb of the Mouse. *Int. J. Radiat. Biol.*, **7**, 113.

SPITALIER, J., BRANDONE, H., AYME, Y., AMALRIC, R., SANTAMARIA, F. & SEIGLE, J. (1977) Cesium Therapy of Breast Cancer. A Five-year Report on 400 Consecutive Patients. *Int. J. Rad. Oncol. Biol. Phys.*, **2**, 231.

SUMMERS, W. C., CLIFTON, K. H. & VERMUND, H. (1964) X-irradiation of the Tumor Bed. I. A Study of the Indirect Actions of Radiation on Transplantable Tumors. *Radiology*, **82**, 691.

TAKAHASHI, M. & KALLMAN, R. F. (1977) Quantitative Estimation of Histological Changes in Subcutaneous Vasculature of the Mouse after X-irradiation. *Int. J. Rad. Oncol. Biol. Phys.*, **2**, 61.

URANO, M. & SUIT, H. D. (1971) Experimental Evaluation of Tumor Bed Effect for C3H Mouse Mammary Carcinoma and for C3H Mouse Fibrosarcoma. *Radiat. Res.*, **45**, 41.